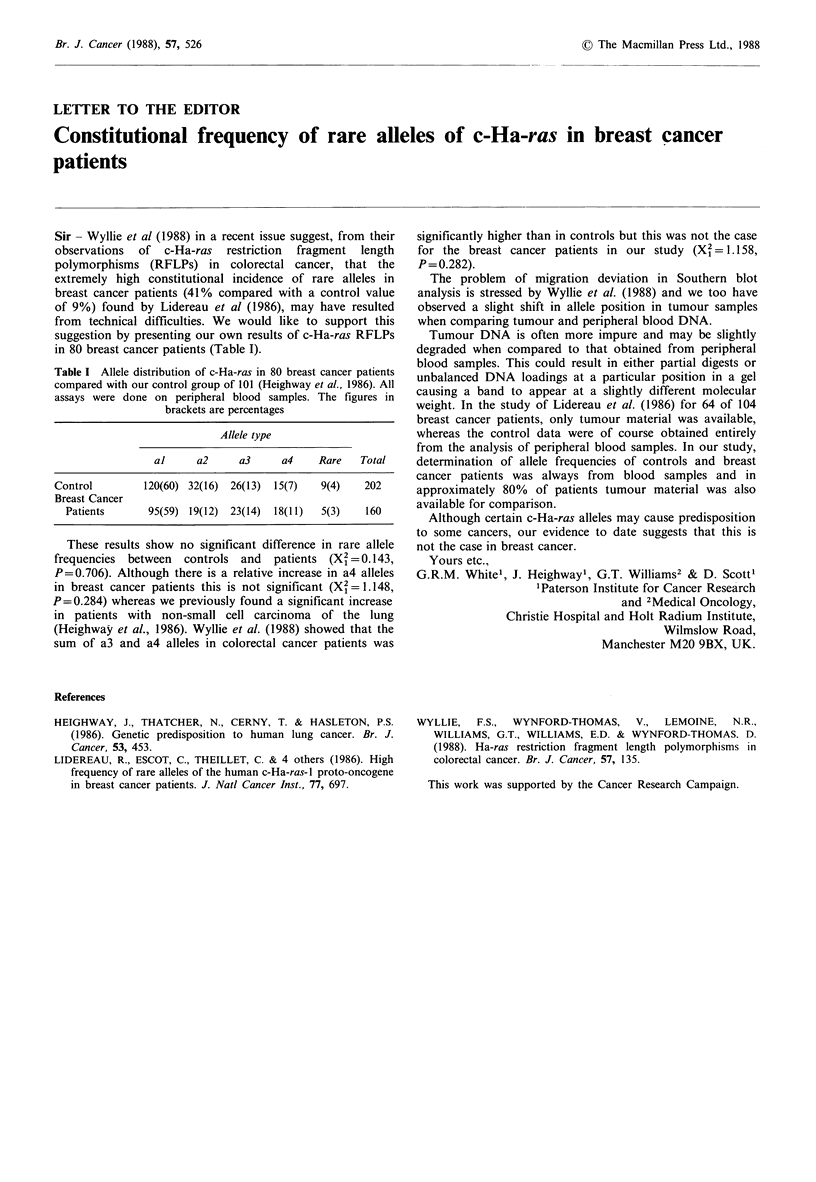# Constitutional frequency of rare alleles of c-Ha-ras in breast cancer patients.

**DOI:** 10.1038/bjc.1988.120

**Published:** 1988-05

**Authors:** G. R. White, J. Heighway, G. T. Williams, D. Scott


 © The Macmillan Press Ltd., 1988

### LETTER TO THE EDITOR

### Constitutional frequency of rare alleles of c-Ha-*ras* in breast cancer patients

**Sir** – Wyllie *et al* (1988) in a recent issue suggest, from their observations of c-Ha-*ras* restriction fragment length polymorphisms (RFLPs) in colorectal cancer, that the extremely high constitutional incidence of rare alleles in breast cancer patients (41% compared with a control value of 9%) found by Lidereau *et al* (1986), may have resulted from technical difficulties. We would like to support this suggestion by presenting our own results of c-Ha-*ras* RFLPs in 80 breast cancer patients (Table I).

**Table I** Allele distribution of c-Ha-*ras* in 80 breast cancer patients compared with our control group of 101 (Heighway *et al.*, 1986). All assays were done on peripheral blood samples. The figures in brackets are percentages

| | *Allele type* | | | | | |
|---|---|---|---|---|---|---|
| | *a1* | *a2* | *a3* | *a4* | *Rare* | *Total* |
| Control | 120(60) | 32(16) | 26(13) | 15(7) | 9(4) | 202 |
| Breast Cancer Patients | 95(59) | 19(12) | 23(14) | 18(11) | 5(3) | 160 |

These results show no significant difference in rare allele frequencies between controls and patients ($X_1^2=0.143$, $P=0.706$). Although there is a relative increase in a4 alleles in breast cancer patients this is not significant ($X_1^2=1.148$, $P=0.284$) whereas we previously found a significant increase in patients with non-small cell carcinoma of the lung (Heighway *et al.*, 1986). Wyllie *et al.* (1988) showed that the sum of a3 and a4 alleles in colorectal cancer patients was significantly higher than in controls but this was not the case for the breast cancer patients in our study ($X_1^2=1.158$, $P=0.282$).

The problem of migration deviation in Southern blot analysis is stressed by Wyllie *et al.* (1988) and we too have observed a slight shift in allele position in tumour samples when comparing tumour and peripheral blood DNA.

Tumour DNA is often more impure and may be slightly degraded when compared to that obtained from peripheral blood samples. This could result in either partial digests or unbalanced DNA loadings at a particular position in a gel causing a band to appear at a slightly different molecular weight. In the study of Lidereau *et al.* (1986) for 64 of 104 breast cancer patients, only tumour material was available, whereas the control data were of course obtained entirely from the analysis of peripheral blood samples. In our study, determination of allele frequencies of controls and breast cancer patients was always from blood samples and in approximately 80% of patients tumour material was also available for comparison.

Although certain c-Ha-*ras* alleles may cause predisposition to some cancers, our evidence to date suggests that this is not the case in breast cancer.

Yours etc.,

G.R.M. White[1], J. Heighway[1], G.T. Williams[2] & D. Scott[1]
[1]Paterson Institute for Cancer Research
and [2]Medical Oncology,
Christie Hospital and Holt Radium Institute,
Wilmslow Road,
Manchester M20 9BX, UK.

**References**

HEIGHWAY, J., THATCHER, N., CERNY, T. & HASLETON, P.S. (1986). Genetic predisposition to human lung cancer. *Br. J. Cancer*, **53**, 453.

LIDEREAU, R., ESCOT, C., THEILLET, C. & 4 others (1986). High frequency of rare alleles of the human c-Ha-*ras*-1 proto-oncogene in breast cancer patients. *J. Natl Cancer Inst.*, **77**, 697.

WYLLIE, F.S., WYNFORD-THOMAS, V., LEMOINE, N.R., WILLIAMS, G.T., WILLIAMS, E.D. & WYNFORD-THOMAS, D. (1988). Ha-*ras* restriction fragment length polymorphisms in colorectal cancer. *Br. J. Cancer*, **57**, 135.

This work was supported by the Cancer Research Campaign.